# Expression of bone morphogenetic protein-2 and its receptors in epithelial ovarian cancer and their influence on the prognosis of ovarian cancer patients

**DOI:** 10.1186/1756-9966-29-85

**Published:** 2010-06-30

**Authors:** Ying Ma, Lin Ma, Quan Guo, Shulan Zhang

**Affiliations:** 1 Department of Obstetrics and Gynecology, Shengjing Hospital of China Medical University, Shenyang 110004, China

## Abstract

**Background:**

To determine the expression of bone morphogenetic protein-2 (BMP-2) and its receptors BMPRIA, BMPRIB, and BMPRII in epithelial ovarian cancer (EOC) and to analyze their influence on the prognosis of ovarian cancer patients.

**Methods:**

Semi-quantitative RT-PCR and western blot were applied to detect the expression of BMP-2 and its receptors BMPRIA, BMPRIB, and BMPRII in EOC, benign ovarian tumors, and normal ovarian tissue at the mRNA and protein levels. Immunohistochemistry was used to determine the expression of BMP-2 and its receptors in 100 patients with EOC to analyze their influence on the five-year survival rate and survival time of ovarian cancer patients.

**Results:**

(1) The mRNA and protein expression levels of BMP-2, BMPRIB, and BMPRII in ovarian cancer tissue were remarkably lower than those in benign ovarian tumors and normal ovarian tissue, while no significant differences in BMPRIA expression level was found among the three kinds of tissues. (2) The five-year survival rate and the average survival time after surgery of EOC patients with positive expression of BMP-2, BMPRIB, and BMPRII were remarkably higher than those of patients with negative expression of BMP-2, BMPRIB, and BMPRII. BMPRIA expression was not associated with the five-year survival rate or with the average survival time of ovarian cancer patients.

**Conclusions:**

BMP-2, BMPRIB, and BMPRII exhibited low expression in EOC tissue, and variation or loss of expression may indicate poor prognosis for ovarian cancer patients.

## Background

Ovarian cancer is the most lethal type of malignant tumors of the female reproductive system, and despite recent developments in diagnosis and treatment techniques, the five-year survival rate for ovarian cancer patients is only 20-40%[1]. The low survival rate is likely due to the lack of early symptoms for this cancer; most patients are diagnosed at an advanced stage and exhibit widespread metastasis. At present, the pathological causes of ovarian cancer are unclear. Thus, it is urgent to investigate and search for novel treatment regimens.

The development of tumors is believed to be a complex process involving several genes and several factors, and more and more influencing factors are emerging. In recent years, researchers have focused on the correlation between bone morphogenetic proteins (BMPs) and the presence of tumors. BMP is a member of the transforming growth factor-β superfamily. Initially, it was thought to induce bone formation and chondrogenesis *in vivo*, and current evidence suggests that it also participates in various biological processes of cells, such as proliferation, differentiation, and apoptosis[2]. BMP signaling is mediated by transmembrane serine/threonine kinases, namely, BMPRI (BMPRIA, BMPRIB) and BMPRII receptors[3]. There are 16 kinds of BMPs, and the majority of studies have focused on BMP-2, which has been shown to play a crucial role in the occurrence and development of breast cancer[4-6], lung cancer[7-11], prostatic carcinoma[12-14], and colon cancer[15,16]. However, the correlation between BMP-2 and ovarian cancer remains unclear. This study was designed to determine the expression of BMP-2 and its receptors in epithelial ovarian cancer, benign ovarian tumors, and normal ovarian tissue and to analyze their influence on the five-year survival rate and average survival time of ovarian cancer patients.

## Methods

### Samples

RT-PCR samples: A total of 29 EOC patients, 32 benign ovarian tumor patients, and 10 patients with normal ovarian tissue were recruited from Shengjing Hospital, which is affiliated with China Medical University, between August 2005 and August 2007.

Western blot samples: A total of 15 EOC patients, 15 benign ovarian tumor patients, and 10 patients with normal ovarian tissue were recruited from Shengjing Hospital, which is affiliated with China Medical University, between August 2005 and August 2007.

Immunohistochemistry samples: One hundred paraffin-embedded specimens of EOC preserved at the Department of Pathology of Shengjing Hospital between January 1997 and August 2001 were included in this study. Specimens were examined for histological grade based on World Health Organization criteria. All EOC patients were grade II and grade III. The tumor stages were determined according to the International Federation of Gynecology and Obstetrics (FIGO) with surgically and cytologically stage performed, all EOC patients had stage III and stage IV. All specimens were fixed with paraformaldehyde, embedded in paraffin, and prepared as serial slices of 4 μm in thickness.

All experiment subjects had complete clinical pathological data and were aged 20-72 years (mean: 50.36 ± 12.30), and there were no significant differences between age groups. No patients received radiotherapy, chemotherapy, biotherapy, or any other operation before surgery for the cancer. Maximal surgical cytoreduction is followed by the standard systemic chemotherapy for these patients. The pathological diagnosis was performed by experts at the Department of Pathology of Shengjing Hospital and the Fourth Hospital affiliated with China Medical University. All samples and clinical data were obtained with the consent from all patients.

### Main reagents

BMP-2 rabbit anti-human polyclonal antibody, SABC immunohistochemical kit, DAB color-developing reagent (Wuhan Boster, China); β-actin rat anti-human polyclonal antibody, BMPRIA rabbit anti-human polyclonal antibody, BMPRIB rabbit anti-human polyclonal antibody, BMPRII goat anti-human polyclonal antibody (Santa Cruz, USA); BMP-2, BMPRIA, BMPRIB, BMPRII and internal reference β-actin primers (Shanghai Sangon, China); Trizol total RNA extraction reagent (Invitrogen, USA); RT kit and PCR amplification reagents (TaKaRa-Dalian, China); alkaline phosphatase-labeled goat anti-rabbit IgG, rabbit anti-goat IgG (Sigma, USA); Bradford protein kit (Nanjing Keygen, China); nitrocellulose filter (Shanghai Gene, China), β-naphthyl acid phosphate, O-dianisidine (tetrazotized; Sigma, USA).

### RT-PCR

In accordance with the instructions for the Trizol total RNA extraction kit, total RNA was extracted from 100 mg specimens, and the ratio of OD_260 _and OD_280 _was 1.8-2.0. The harvested RNA was diluted to a concentration of 1 μg/ul, packaged, and preserved at -70°C. The conditions for the first round of RT synthesis of cDNA were as follows: 42°C for 30 min, 99°C for 5 min, and 5°C for 5 min. PCR reaction conditions were as follows: for BMP-2, BMPRIA, BMPRII, and β-actin: 94°C for 2 min, 94°C for 30 s, 55°C for 30 s, and 72°C for 45 s for a total of 30 cycles, then 72°C for 7 min; for BMPRIB: 94°C for 2 min, 94°C for 30 s, 53°C for 30 s, and 72°C for 45 s, for a total of 30 cycles, then for 72°C for 7 min. Primer sequences were as follows:

BMP-2:

5'-CCAACCATGGATTCGTGGTG-3',

5'- GGTACAGCATCGAGATAGCA-3'

BMPRIA:

5'-AATGGAGTAACCTTAGCACCAGAG-3',

5'-AGCTGAGTCCAGGAACCTGTAC-3'

BMPRIB:

5'- GCAGCACAGACGGATATTGT-3',

5'- TTTCATGCCTCATCAACACT-3'

BMPRII:

5'-ACGGGAGAGAAGACGAGCCT-3',

5'-CTAGATCAAGAGAGGGTTCG-3';

β-actin:

5'-GTGGGGCGCCCCAGGCACCA-3',

5'-CTCCTTAATGTCACGCACGATTTC-3'

After 1.5% agarose gel electrophoresis with 1 μg/μl ethidium bromide dye, RT-PCR products were observed with a GIS-2020 gel scanning image analytical system. By using DNA Marker DL2000 as the standard molecular weight and β-actin as an internal reference, the ratio of BMP-2, BMPRIA, BMPRIB, BMPRII, and β-actin was calculated. RT-PCR products were semiquantitatively analyzed.

### Western blot

In accordance with the instructions for the total protein extraction kit, total protein was extracted from 100 mg specimens. Protein concentrations were assayed by the Bradford method, and specimens were adjusted to the same protein concentration, packaged, and preserved at -70°C for later use. With a prestained marker serving as an index, the necessary gels were selected after polyacrylamide gel electrophoresis was performed, and a nitrocellulose filter was used for the transfer print. The primary antibody concentration was 1:100 and the secondary antibody was 1:2,000. By using alkaline phosphatase coloration, the protein hybridization band was scanned with a GIS-2020 digital image analysis system and the absorbance value was assayed. The ratios of BMP-2, BMPRIA, BMPRIB, BMPRII, and β-actin were calculated for the semiquantitative analysis.

### Immunohistochemistry

Paraffin slices were treated according to the SABC immunohistochemical kit, and results were analyzed using a double-blind method. Five high-power fields (×400) were selected at random, and two pathologists evaluated scores independently. PBS, instead of the primary antibody, was used as negative control, and specimens were scored according to the intensity of the dye color and the number of positive cells. The intensity of the dye color was graded as 0 (no color), 1 (light yellow), 2 (light brown), or 3 (brown), and the number of positive cells was graded as 0 (<5%), 1 (5-25%), 2 (25-50%), 3 (51-75%), or 4 (>75%). The two grades were added together and specimens were assigned to one of 4 levels: 0-1 score (-), 2 scores (+), 3-4 scores (++), more than 5 scores (+++). The positive expression rate was expressed as the percent of the addition of (++) and (+++) to the total number.

### Statistical analysis

Statistical analysis was performed with SPSS version 11.0 software, and *P *< 0.05 was considered to be statistically significant. Statistical tests used included the chi square test and analysis of variance.

## Results

### RT-PCR

**Figure 1:**
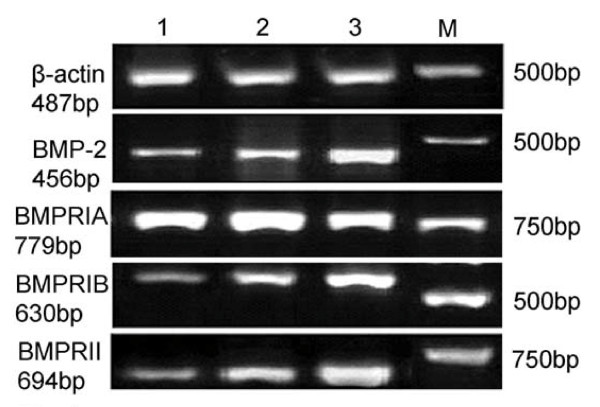
**The mRNA expression of BMP-2 and its receptors detected by RT-PCR 1: Ovarian cancer tissue; 2: Benign ovarian tumor tissue; 3: Normal ovarian tissue; M: Marker**.

The mRNA expression levels of BMP-2, BMPRIB, and BMPRII in ovarian cancer tissues was significantly lower than those in benign ovarian tumors or normal ovarian tissue. No significant differences in BMPRIA mRNA expression level were observed among the three kinds of tissue (Table 1 and Figure 1). The relative content of the proteins was expressed as mean ± standard deviation (SD).

**Table 1:** Relative content of mRNA of BMP-2 and its receptors in ovarian tissue

	BMP-2	BMPRIA	BMPRIB	BMPRII
Ovarian cancer	0.875 ± 0.136	1.525 ± 0.158	0.808 ± 0.137	0.834 ± 0.138
Benign ovarian tumor	1.409 ± 0.089	1.569 ± 0.198	1.173 ± 0.143	1.016 ± 0.119
Normal ovarian tissue	1.598 ± 0.082	1.455 ± 0.176	1.234 ± 0.162	1.273 ± 0.179
P value	0.001	0.680	0.001a	0.001

^a^*P *= 0.548, comparison between benign ovarian tumor and normal ovarian tissue.

### Western blot

**Figure 2:**
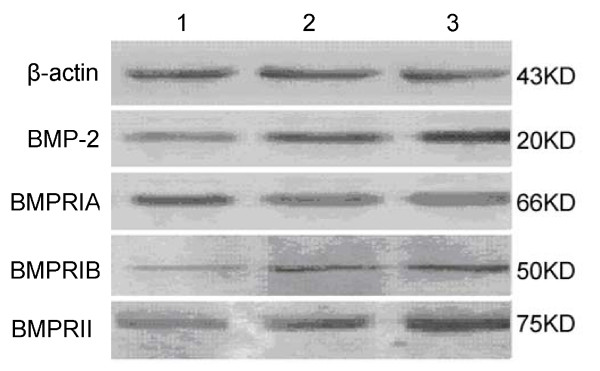
**The protein expression of BMP-2 and its receptors detected by western blot 1: Ovarian cancer tissue; 2: Benign ovarian tumor tissue; 3: Normal ovarian tissue**.

The relative content of the proteins BMP-2, BMPRIB, and BMPRII in ovarian cancer tissue was significantly lower than those in benign ovarian tumors or normal ovarian tissue. No significant differences in BMPRIA protein expression level were observed among the three kinds of tissue (Table 2 and Figure 2). The relative content was expressed as mean ± standard deviation (SD).

**Table 2:** Relative content of BMP-2 protein of BMP-2 and its receptors in ovarian tissues

	BMP-2	BMPRIA	BMPRIB	BMPRII
Ovarian cancer	0.805 ± 0.105	0.951 ± 0.101	0.816 ± 0.108	0.867 ± 0.119
Benign ovarian tumor	0.958 ± 0.103	0.911 ± 0.113	0.905 ± 0.115	0.974 ± 0.097
Normal ovarian tissue	0.975 ± 0.082	1.026 ± 0.099	1.029 ± 0.087	1.077 ± 0.103
P value	0.019	0.361	0.042	0.043

### Immunohistochemistry

**Figure 3:**
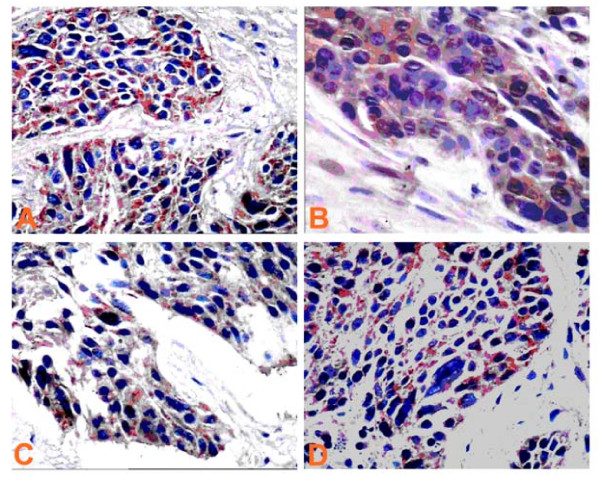
**Expression of BMP-2, BMPRIA, BMPRIB, and BMPRII in epithelial serous ovarian cancer detected by immunohistochemistry (×400) A: BMP-2, B: BMPRIA, C: BMPRIB, D: BMPRII**.

Positively stained BMP-2 and its receptors BMPRIA, BMPRIB, and BMPRII were mainly located in the cytoplasm of ovarian cancer cells and appeared as light brown and brown particles (Figure 3).

Retrospective analysis of follow-up visits of patients showed that the total five-year survival rate of 100 patients was 32% with a mean survival time of 32.42 ± 22.62 months. The five-year survival rate after surgery of ovarian cancer patients with positive expression of BMP-2, BMPRIB, and BMPRII was remarkably higher than that of patients with negative expression of BMP-2, BMPRIB, and BMPRII. BMPRIA expression was not associated with the five-year survival rate of ovarian cancer patients (Table 3).

**Table 3:** Correlation of the expression of BMP-2 and its receptors with survival rate and survival time of ovarian cancer patients

	BMP2	BMPRIA	BMPRIB	BMPRII
Positive expression rate (%)	62	49	62	53
Negative expression rate (%)	38	51	38	47
Five-year survival rate of positive cases	40.32	32.66	41.94	41.51
Five-year survival rate of negative cases	18.42	31.37	15.79	21.28
*P *value	0.023	0.891	0.007	0.030
Survival time of negative cases	37.27 ± 21.46	33.71 ± 21.95	37.66 ± 22.54	37.21 ± 22.10
Survival time of negative cases	24.50 ± 22.47	31.18 ± 23.40	23.87 ± 20.25	27.02 ± 22.20
*P *value	0.006	0.577	0.003	0.024

## Discussion

In 1965, Urist successfully induced heterotopic bone formation by grafting decalcified bovine bone into muscles and skin[17]. Accordingly, we conclude that some substance in bone matrix is capable of inducing bone formation, namely BMP. BMP can differentiate mesenchymal cells into osteoblasts, plays various roles during embryonic development, and is of crucial importance to the nervous system, hematopoietic cells, the heart and liver, etc. BMP cannot act without its receptors, namely, BMPRI (BMPRIA and BMPRIB) and BMPRII, which are located on chromosomes 10q23, 4q22-24, and 2q33-34. BMPRIA mediates growth stimulation signals, and BMPRIB transfers growth inhibition signals[3]. BMPs bind with type II receptors first, after which the type II receptor phosphorylates the type I receptor. The receptor-ligand complex phosphorylates the Smad system, and then the complex shifts into the cell nucleus and is involved in gene transcription, thus transferring the BMP signal to the target gene.

At present, there are 16 known BMPs, and the majority of research has focused on BMP-2. In 1988, Wozney screened a gene named hBMP-2 from human U-20S cell cDNA based on a bovine BMP amino acid sequence[18]. The hBMP-2 cDNA was 1188 bp in length, encoded 396 amino acids, and was located on chromosome 20 in the p21 region.

BMP-2 plays an important physiological role in various tissues throughout the body and has been shown to be expressed in tumor tissues. Moreover, its effects vary depending on the tissue. For example, studies have demonstrated that BMP-2 and its receptors are expressed in breast cancer[19], colon cancer[15], gastric cancer[20] and that its expression may be associated with the biological behavior of the tumor. In vitro trials have confirmed that BMP-2 can inhibit the growth of some tumors. Conversely, other research has suggested that BMP-2 can stimulate the growth of tumor cells in vitro, such as lung cancer[9,10] and prostatic carcinoma[21]. There are only a few reports on the correlation of BMP-2 and ovarian cancer. For instance, Kiyozuka [22] and Le Page [23] both detected the expression of BMP-2 in ovarian cancer tissues, and Kiyozuka further confirmed that BMP-2 was involved in the formation of serous ovarian cancer psammoma bodies. Soda[16] has reported that BMP-2 can inhibit the growth of cancer cell clones in 2 of 15 ovarian cancer patients, but no study has investigated the influence of BMP-2 on prognosis for ovarian cancer patients or the underlying mechanisms behind its role in the development of ovarian cancer.

In this study, BMP-2 was shown to be expressed in ovarian cancer, benign ovarian tumors, and normal ovarian tissue, and its expression in ovarian cancer was clearly lower than the latter two. This evidence suggests that the BMP-2 gene is likely expressed in normal ovarian tissue, where it acts as a protective factor. Thus, variation or loss of its expression may promote the development of ovarian cancer. The BMP-2 receptors BMPRIA, BMPRIB, and BMPRII were also expressed in all three types of tissue, and the expression levels of BMPRIB and BMPRII in ovarian cancer tissue was significantly lower than those in benign ovarian tumors and normal ovarian tissue, although the difference in the BMPRIA expression level between the different tissues was not significant. This suggests that BMP-2 may act through its receptors, BMPRIB and BMPRII, in ovarian cancer. Previous studies have shown that BMPRIA mediates growth stimulation signals, while BMPRIB transfers growth inhibition signals. Our evidence suggests that the weakening of the inhibitory effect of BMP-2 and BMPRIB may promote the development of ovarian cancer. It is possible that BMPRIA has no correlation with the development of ovarian cancer. That is, the development of ovarian cancer is not due to the stimulatory effect of BMPRIA.

In order to investigate the influence of BMP-2 on the prognosis of ovarian cancer patients, 100 patients were followed up after their surgery. Their five-year survival rate was 32%, a rate that is consistent with other published reports. Immunohistochemistry results demonstrated that ovarian cancer patients with positive expression of BMP-2, BMPRIB, and BMPRII exhibited remarkably higher five-year survival rates and average survival rates than patients with negative expression of BMP-2, BMPRIB, and BMPRII. BMPRIA showed no association with five-year survival rate or with survival time of ovarian cancer patients. BMP-2, BMPRIB, and BMPRII may play a part in the occurrence and development of ovarian cancer, and the variation or loss of expression of BMP-2, BMPRIB, and BMPRII may be an indicator of poor prognosis for ovarian cancer patients. Further studies conducted with larger sample sizes are needed to confirm this association.

Our study suggests that BMP-2 and its receptors BMPRIB and BMPRII are likely to be involved in the development of ovarian cancer, and attenuation or loss of expression may result in or indicate poor prognosis for ovarian cancer patients. However, the specific pathway and mechanisms driving this effect need further study, if novel treatments for ovarian cancer are to be achieved through better understanding of its pathogenesis.

## Conclusions

BMP-2, BMPRIB, and BMPRII exhibited a low expression in EOC tissue. The variation or loss of expression of these markers may indicate poor prognosis for ovarian cancer patients.

## Abbreviation list

EOC: epithelial ovarian cancer; BMPs: bone morphogenetic proteins; BMP-2: bone morphogenetic protein-2; BMPRIA: bone morphogenetic protein-2 receptor IA; BMPRIB: bone morphogenetic protein-2 receptor IB; BMPRII: bone morphogenetic protein-2 receptor II.

## Competing interests

The authors declare that they have no competing interests.

## Authors' contributions

YM carried out RT-PCR and Western blot, performed the statistical analysis and wrote the paper. LM participated in the design of the study and contributed with drafting the manuscript. QG carried out the immunohistochemistry studies. SLZ participated in coordination. All authors read and approved the final manuscript.
